# RETRACTION: The Influence of Different Peritoneal Dialysis Techniques on Wound Infection in Patients With Peritoneal Dialysis Tube

**DOI:** 10.1111/iwj.70337

**Published:** 2025-03-17

## Abstract

**RETRACTION:** LiuT. and WangY. , “The Influence of Different Peritoneal Dialysis Techniques on Wound Infection in Patients With Peritoneal Dialysis Tube,” International Wound Journal 21, no. 1 (2024): e14352, 10.1111/iwj.14352.37622537 PMC10781592

The above article, published online on 25 August 2023, in Wiley Online Library (http://onlinelibrary.wiley.com/), has been retracted by agreement between the journal Editor in Chief, Professor Keith Harding; and John Wiley & Sons Ltd. Following an investigation by the publisher, all parties have concluded that this article was accepted solely on the basis of a compromised peer review process. The editors have therefore decided to retract the article. The authors did not respond to our notice regarding the retraction.

**RETRACTION:** T. Liu and Y. Wang , “The Influence of Different Peritoneal Dialysis Techniques on Wound Infection in Patients With Peritoneal Dialysis Tube,” International Wound Journal 21, no. 1 (2024): e14352, 10.1111/iwj.14352.37622537 PMC10781592

The above article, published online on 25 August 2023, in Wiley Online Library (http://onlinelibrary.wiley.com/), has been retracted by agreement between the journal Editor in Chief, Professor Keith Harding; and John Wiley & Sons Ltd. Following an investigation by the publisher, all parties have concluded that this article was accepted solely on the basis of a compromised peer review process. The editors have therefore decided to retract the article. The authors did not respond to our notice regarding the retraction.

